# Genetic mismatch affects the immunosuppressive properties of mesenchymal stem cells *in vitro *and their ability to influence the course of collagen-induced arthritis

**DOI:** 10.1186/ar3916

**Published:** 2012-07-19

**Authors:** Catherine Sullivan, J Mary Murphy, Matthew D Griffin, Ryan M Porter, Christopher H Evans, Cathal O'Flatharta, Georgina Shaw, Frank Barry

**Affiliations:** 1 Regenerative Medicine Institute, National University of Ireland Galway, University Road, Galway, Ireland; 2 Centre for Advanced Orthopaedic Studies, Beth Israel Deaconess Medical Centre, 330 Brookline Ave., RN 115, Boston , MA 02215, USA

## Abstract

**Introduction:**

The immunological and homing properties of mesenchymal stem cells (MSCs) provide a potentially attractive treatment for arthritis. The objective of this study was to determine effects of genetic disparity on the immunosuppressive potential of MSCs *in vitro *and *in vivo *within collagen induced arthritis (CIA).

**Methods:**

The ability of DBA/1, FVB and BALB/c MSC preparations to impact the cytokine release profile of CD3/CD28 stimulated DBA/1 T cells was assessed *in vitro*. The effect of systemically delivered MSCs on the progression of CIA and cytokine production was assessed *in vivo*.

**Results:**

All MSC preparations suppressed the release of TNFα and augmented the secretion of IL-4 and IL-10 by stimulated DBA/1 T-cells. However, assessment of the ratio of IFNγ to IL-4 production indicated that the more genetically distant BALB/c MSCs had significantly less immunosuppressive capacity. Systemic delivery of BALB/c MSC resulted in an exacerbation of CIA disease score *in vivo *and a higher erosive disease burden. This was not seen after treatment with syngeneic or partially mismatched MSCs. An increase in serum levels of IL-1β was observed up to 20 days post treatment with allogeneic MSCs. An initial elevation of IL-17 in these treatment groups persisted in those treated with fully mismatched BALB/c MSCs. Over the course of the study, there was a significant suppression of serum IL-17 levels in groups treated with syngeneic MSCs.

**Conclusions:**

These data demonstrate a significant difference in the immunosuppressive properties of syngeneic and allogeneic MSCs *in vitro *and *in vivo*, which needs to be appreciated when developing MSC based therapies for inflammatory arthritis.

## Introduction

Rheumatoid Arthritis (RA) is characterized by systemic inflammation and local synovitis with pannus formation. The inflamed synovium is populated with CD4+ T lymphocytes, B cells, macrophages and synovial like fibroblasts which elaborate pathophysiologically important cytokines including TNFα, IL-1, IL-4, IL-10 and IL-17 [1-3]. While numerous cells are involved in disease the ongoing activation of T cells is central in perpetuating tissue inflammation and damage through stimulation of mesenchymal cells, including chondrocytes and fibroblasts [2]. Early studies demonstrating the central role of TNFα have been translated into the development of blocking drugs which have revolutionized the treatment of RA [4]. Despite recent advances and wide availability of anti-TNF agents, a considerable number of patients remain refractory to treatment [5-7]. Other therapeutic strategies include B cell depletion, inhibition of T cell co-stimulation and anti IL-6 receptor antibodies [8,9].

While TNFα produced by macrophages is at the center of a complex cytokine network in inflammatory arthritis, it is well established that CD4+ T cells play an important role in orchestrating and maintaining this immune response. The production of Th1 cytokines in animal models is associated with arthritis induction and inflammation and collagen induced arthritis (CIA) is a Th1 driven disease, while Th2 cytokines are found during disease remission [10]. However, the role of IFNγ in CIA is complex with a peak in early disease and evidence of a disease limiting role in late disease decreasing IL-17 production and osteoclast precursors while increasing the activity of T regulatory cells [11]. IL-17 is produced by a subset of memory CD4+ T lymphocytes termed Th17 cells and plays a critical role in CIA, having a synergistic effect with TNFα in promoting poor disease outcome [12-14]. IL-1β has also been implicated in both RA and CIA; it is known to have a pro-inflammatory role in CIA which is independent of IL-17 and inhibition with an IL-1 receptor antagonist results in amelioration of arthritis [15,16]. The role of anti-inflammatory cytokines in CIA is less clear; however, IL-4 can inhibit production of TNFα and reduce T cell proliferation [2].

There is considerable interest in bone marrow derived mesenchymal stem cells (MSCs) which have the capacity to differentiate into cells of the connective tissue compartment including bone, cartilage and fat. Both mouse and human MSCs are known to have immunosuppressive effects on T and B cells *in vitro *[17-20] and this is likely to be an important aspect of their mode of action. MSCs can inhibit proliferation of CD4^+ ^and CD8^+ ^T cells in a dose-dependent manner independently of major histocompatibility complex (MHC) matching with reduced expression of activation markers [21,22]. The mechanisms underlying this immune suppression are not fully understood and may be mediated by transforming growth factor-β, hepatocyte growth factor, IL-10 or prostaglandin-E2 produced by the cells [21,23,24]. Additionally, MSC immune regulation may be mediated through secondary effects on other cells, such as increased TNFα and IL-10 production by dendritic cells and decreased IFNγ production by Th1 cells [25-27].

*In vitro *studies suggest that the immunosuppressive properties of MSCs may be useful for in *vivo *treatment of inflammatory arthritis [28]. MSCs from healthy donors can inhibit type II collagen (CII) stimulated proliferation of T cells from arthritis patients [29]. In addition, adipose derived stem cells (ASC) can exert profound suppressive responses on CII-reactive T cells by suppressing CII-induced T cell proliferation, inhibiting inflammatory cytokine production and stimulating production of IL-10 by monocytes and T cells. Co-culture of ASCs with T cells induced the generation of antigen-specific T regulatory cells and ASCs were also shown to inhibit production of inflammatory factors by activated synovial cells involved in the destruction of cartilage and bone [30]. Overall, it is clear that MSCs exert immunosuppressive effects on several classes of lymphocytes through mechanisms that may be exploited in a clinical setting. As MSCs may migrate to sites of injury *in vivo *it is feasible to suggest that targeting of the cells to inflamed joints might have a therapeutic effect on local arthritis through MSC-mediated immunosuppression [31,32]. An additional property of MSCs is their status as immune privileged cells with low expression of MHC II [33,34] and an absence of co-stimulatory molecules [21,35]. This raises the prospect of allogeneic cell therapy in a variety of disease models. However, translation of compelling *in vitro *data to pre-clinical studies of arthritis has resulted in conflicting data [30,36-41]. Studies using either single or multiple injections of MSCs have shown varying degrees of disease amelioration or exacerbation. MSCs have been delivered at various time points, including at disease induction or during established disease, and generally at doses of 1 × 10^6 ^cells, although in some cases up to 5 × 10^6 ^cells. In an effort to shed further light on the disease-modifying effects of allogeneic MSC transplantation, we looked at the effects of genetic disparity between systemically delivered MSCs and the host recipient. We hypothesized that the degree of mismatch between donor and host may exert a profound effect on their immunosuppressive properties. We studied this *in vitro *by examining the interactions between MSCs and T cells when the cells were syngeneic, partially mismatched or fully mismatched. We achieved this by examining the effects of MSCs from DBA/1 mice on T cells from the same strain (syngeneic), from FVB mice (partially mismatched) and from BALB/c mice (fully mismatched). For *in vivo *studies we considered it important to establish if the genetic background affected the ability of a single injection of MSCs at the time of onset of clinical disease to alter disease progression in CIA.

## Materials and methods

### Mesenchymal stem cell isolation and characterization

MSCs were isolated from the bone marrow of eight- to ten-week old BALB/c and FVB mice as described previously [42]. For DBA/1 mice collagenase digestion of the marrow was carried out before MSC expansion in complete expansion medium (Iscove's modified eagle's medium, 9% horse and 9% fetal bovine serum (FBS), 1% penicillin and streptomycin (P/S), 1% L-glutamine; CEM). It has been demonstrated that murine bone marrow derived MSCs isolated using collagenase digestion retain the characteristic properties of MSC [43]. Briefly, mononuclear cells from two femurs were plated in a T-175 flask. Sufficient fibroblastic-like cells were obtained at the end of P1 to replate at 50 cells/cm^2 ^and 4,500 cells/cm^2 ^were plated for subsequent passages with about a 10-fold increase in cell number by confluency (seven days). Expression of cell surface antigens was quantified using flow cytometry. Briefly, 200,000 cells were used per antibody staining. After blocking in PBS with 2% FBS, 1% rat serum for 30 minutes on ice, cells were centrifuged at 400 g for 5 minutes and resuspended in 100μl of the appropriate antibody diluted in PBS with 2% FBS. CD73, CD34 and CD31 antibodies were used at 1:200, Sca1, CD105, CD44 and CD29 at 1:400 and CD45 at 1:500. Cells were incubated on ice for 30 minutes in the dark, washed in PBS with 2% FBS and resuspended in 200μl prior to analysis on the BD FACS Canto flow cytometer. All antibodies were from eBioscience (Hatfield, UK) except for anti-CD45 (Becton Dickinson, Franklin Lakes, NJ, USA). All MSC preparations were stimulated to undergo adipogenesis, osteogenesis and chondrogenesis as described previously using CEM as the basal medium for osteogenesis and adipogenesis [44]. Adipogenesis was confirmed with Oil Red O staining for lipid vacuoles. Osteogenesis was assessed using quantitation of calcium with the Calcium Liquicolour Kit (StanBio, Boerne, TX, USA) and mineral deposition visualized using alizarin red. For chondrogenesis cell pellets were digested with papain and glycosaminoglycan (GAG) was determined using the dimethylmethylene blue assay. Histological sections (5 µM) of formalin-fixed pellets were stained for the presence of GAG using toluidine blue.

### Collagen induced arthritis

All animal work was carried out with approval from the local Institutional Animal Care and Use Committee. Male mice seven- to nine-weeks old were used for all arthritis induction as female DBA/1 mice are not susceptible to CIA. Mice were obtained from a commercial supplier and housed in groups of two or three; room temperature and food, water supply and general health were assessed daily. There were 12 mice included in each CII treatment group. CIA was induced in male DBA/1 mice by immunization with 50 µL bovine bovine CII emulsified in 50 µL complete Freund's adjuvant followed by a booster immunization in incomplete adjuvant at 21 days. MSCs from eight- to ten-week old mice at passage four to six were delivered intravenously on day 21 via the lateral tail vein; 1 × 10^6 ^MSCs were suspended in 100 µl of PBS and used within 30 minutes of preparation. Cell counts using trypan blue exclusion of dead cells confirmed >95% cell viability for MSCs harvested and stored by these methods for all three strains (results not shown). A control group received PBS. Clinical scores were calculated based on the paw thickness across the metatarsophalangeal joints and dactylitis as follows: paw <2 mm = 0 points, paw 2 to 3 mm = 2 points, paw 3 to 4 mm = 3 points, paw 4 to 5 mm = 4 points, paw 5 to 6 mm = 5 points, swollen wrist or ankle = 3 points, swollen digit = 1 point. The scoring system ensured that disease activity reflected by dactylitis only was not overlooked and avoided reliance on measurement in increments <1 mm and subjective assessment of erythema. The clinical score was assessed every 24 hours. Blood was collected and serum cytokine levels assayed using a Bio-Plex 200 cytokine array. Knees were fixed in formalin, processed, paraffin-embedded, sectioned and stained with a modified Mallory stain [45].

#### *In vitro *T cell stimulation studies

Spleens and submandibular lymph nodes from DBA/1 mice were harvested, placed in ice-cold PBS/10% FBS, shredded and passed through a 70 µm mesh. A combination of splenic and nodal cells were harvested to optimize cell numbers and approximately 20 × 10^6 ^cells were isolated per mouse; for each experiment cells from three DBA/1 mice were mixed. The resulting single cell suspension was centrifuged at 120 g for 10 minutes and resuspended in lysis buffer (Invitrogen, Dublin, Ireland). After three minutes incubation at room temperature, cold PBS/2% FBS was added and the sample centrifuged for 10 minutes. Cells were washed in PBS/2% FBS and either resuspended in medium (HG-DMEM, 10% FBS, 2% P/S, 1% HEPES, 12 µM L-glutamine and 1% non essential amino acids) or used for T cell isolation.

CD4^+ ^T cells were isolated by positive selection using murine CD4 Dynabeads (Miltenyi Biotech, Bergisch Gladbach, Germany). Cells were resuspended in MACS buffer with CD4 microbeads and CD4^- ^cells were separated on an octoMACS magnetic separator (Miltenyi Biotech) according to the manufacturer's instructions. The CD4^+ ^cells were resuspended in DMEM/10% FBS and stimulated with anti-CD3/anti-CD28 beads (1:1 ratio) (Dynabeads mouse T cell expander, Invitrogen) for 24 hours in 96-well tissue culture plates. The activated T cells were washed and 1 × 10^5 ^T cells were replated with adherent MSCs (passage four to six, six donor mice), at an MSC:CD4 cell ratio of 1:10 or 1:1, and re-stimulated by the same method. The re-activation of T cells at the time of addition to MSC cultures was designed to mimic the *in vivo *scenario where mice received the booster immunization which triggers overt inflammation at the time of MSC delivery. Culture supernatants were collected at 24 hours for determination of cytokine concentrations by ELISA (eBioscience Mouse Th1/Th2 and Mouse TNFα ELISA Sets).

### Statistics

Statistical analysis was performed using StatsDirect® software. In all instances the Shapiro Wilks test was used to confirm a normal distribution. For direct comparisons t-tests or Mann Whitney tests were used where appropriate for parametric/non-parametric data, respectively. Comparison between groups was carried out using analysis of variance (ANOVA) with post-hoc Tukey testing.

## Results

### MSC isolation

**Figure 1:**
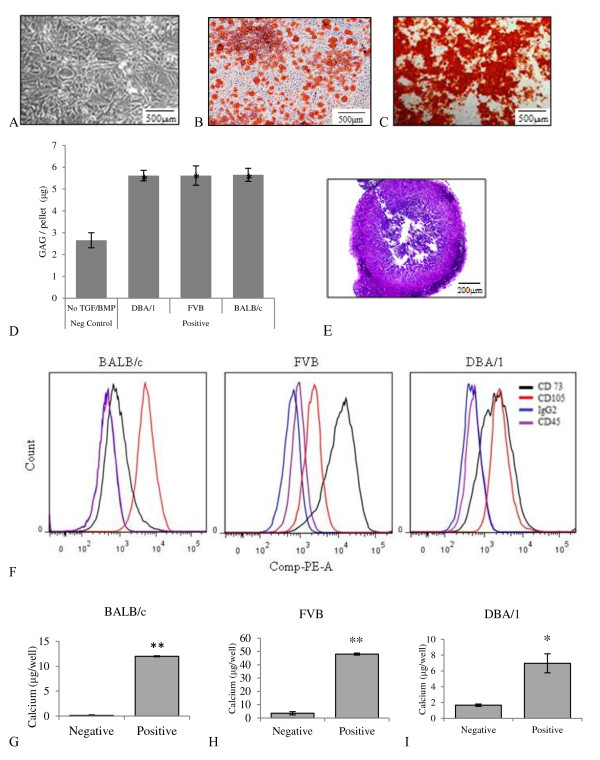
**Mouse MSC characteristics**. Representative images of BALB/c MSCs showing **A**, fibroblastic morphology of MSCs, **B**, adipogenesis with fat vacuoles stained with Oil Red O and **C**, osteogenesis with Alizarin Red staining (scale 500 μm). **D**, proteoglycan (GAG) per 10^5 ^cell pellet demonstrating chondrogenic potential in the presence of TGFβ3 and BMP2. Analysis was performed in triplicate pellets; * *P *<0.05 (Mann Whitney U testing). **E**, histological confirmation of chondrogenesis by BALB/c MSC using toluidine blue staining (scale 200μm). **F**, flow cytometric analysis of BALB/c, FVB and DBA/1 MSCs showing them to be positive for CD73 and CD105, and negative for CD45. **G, H, I**, calcium deposition demonstrating osteogenesis; *P <0.05, **P <0.001 (unpaired T test). Data represents mean ± standard deviation of triplicate cultures. BMP, bone morphogenetic protein; GAG, glycosaminoglycan; MSC, mesenchymal stem cells; TGFβ, transforming growth factor beta.

MSCs were isolated from bone marrow of DBA/1, FVB and BALB/c mice. DBA/1 and FVB mice carry the MHC H2^q ^haplotype while BALB/c mice carry the H2^d ^haplotype. All three MSC strains had a characteristic fibroblastic morphology and were differentiated under appropriate conditions into chondrocytes, adipocytes and osteocytes. Although adipogenesis and chondrogenesis were similar between all three strains, FVB cells had the highest osteogenic potential. All three MSCs preparations were over 95% positive for CD29 and CD44. Detection of CD105 and Sca 1 varied between strains as has been shown previously [42] with approximately 17% of BALB/c MSCs positive for CD73. All three strains of MSCs were negative for CD45, CD34 and CD31 (Figure 1, Table 1) [20,35,42,46]. Cells also lacked expression of MHC II (results not shown).

**Table 1:** Bone marrow stromal cells from all three mouse strains had characteristics of mesenchymal stem cells in terms of morphology, differentiation capacity and cell surface markers.

Mouse Strain	DBA/1	FVB	BALB/c
MHC H2 Haplotype	H2^q^	H2^q^	H2^d^
Morphology	Fibroblastic	Fibroblastic	Fibroblastic
Adipogenesis	Positive	Positive	Positive
Osteogenesis^a^ (μg Ca/well)	6.9 **± **1.6	48.0 **± **0.9	11.9 **± **0.2
Chondrogenesis^a^ (μg GAG/pellet)	5.6 **± **0.3	5.6 **± **0.5	5.6 **± **0.3
CD73^b^	64.8	97	17.2
CD105^b^	82	49.5	97.9
Sca 1^b^	89.9	67.5	53
CD29	99.2	99.4	96.2
CD44	95.1	98.7	99.7
CD45^b^	0.6	4	0.6
CD34^b^	0.4	0.4	0.8
CD31^b^	0.4	0.5	1.3

^a^Data represents mean ± standard deviation of triplicate assays; ^b ^% positive cells; GAG, glycosaminoglycan; MHC, major histocompatibility complex.

### *In vitro *cytokine T cell studies

**Figure 2:**
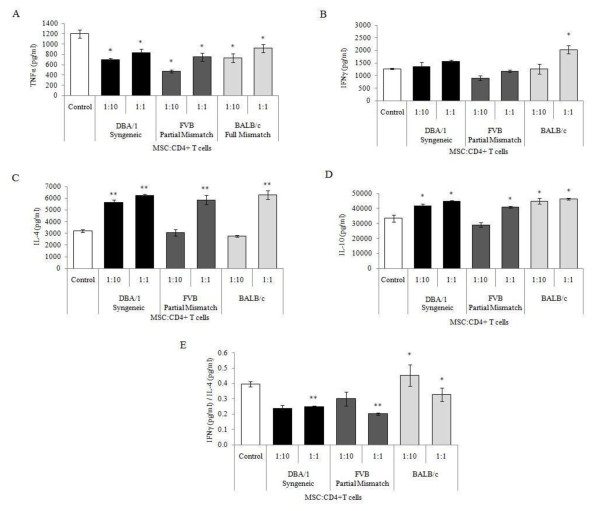
**The effect of MSCs on cytokine production by CD4+ T cells**. Control groups were CD4+ DBA/1 T cells stimulated with CD3/CD28 beads. DBA/1 (syngeneic), FVB (partially mismatched) or BALB/c (fully mismatched) MSCs were co-cultured with DBA/1 CD4+ T cells at a ratio of either 1:10 or 1:1. **A**, Co-culture with MSCs decreased TNFα secretion compared to controls independently of the genetic background of the MSC, *P <0.05 (2-way ANOVA). **B**, BALB/c MSCs increased IFNγ production compared to controls at the ratio of 1:1, *P <0.05 (2-way ANOVA). **C**, DBA/1 MSCs augmented IL-4 production by CD4+ T cells at both ratios. This was also seen with FVB and BALB/c MSCs at the ratio of 1:1, **P <0.01 (2-way ANOVA). **D**, With the exception of FVB MSCs at a ratio of 1:10, the addition of MSCs resulted in an increase in IL-10 production, *P <0.05 (2-way ANOVA). **E**, IFNγ:IL-4 was taken to represent Th1:Th2 cytokine production by stimulated T cells. Co-culture with DBA/1 or FVB MSCs (1:1) significantly suppressed this ratio compared to controls, **P <0.01 (2-way ANOVA). This was not seen when T cells were co-cultured with BALB/c MSCs. The Th1:Th2 ratio of T cells co-cultured with BALB/c MSCs was significantly higher than either the syngeneic or partially mismatched MSC groups, *P <0.05 (2-way ANOVA). Throughout n = 3, data represents mean ± standard deviation. ANOVA, analysis of variance; IFNγ, interferon gamma; IL-10, interleukin 10; IL-4, interleukin 4; MSC, mesenchymal stem cells; n, number; Th, T helper cell; TNFα, tumor necrosis factor alpha.

TNFα and IFNγ were detected in the supernatant of CD4^+ ^T cells 24 hours post-stimulation with CD3/CD28 beads. Only trace levels were measured in the supernatants from unstimulated T cells with and without MSCs, and cultures containing only MSCs (results not shown). The secretion of TNFα by stimulated CD4^+ ^T cells was significantly suppressed by all MSC preparations at ratios of 1:10 and 1:1 (Figure 2A). Syngeneic (DBA/1) and partially mismatched (FVB) MSCs did not significantly affect IFNγ secretion by DBA/1 CD4^+ ^T cells at 24 hours (Figure 2B). Similarly, fully mismatched BALB/c MSCs did not alter IFNγ levels at a ratio of 1:10; however, there was a significant increase in IFNγ production by T cells co-cultured with mismatched MSCs at the higher ratio of 1:1 (Figure 2B).

IL-4 secretion by stimulated CD4^+ ^T cells was detected at 24 hours. This was significantly increased in cultures with MSCs from each genetic background at the 1:1 ratio. With a lower MSC to CD4^+ ^T cell ratio of 1:10 only syngeneic MSCs increased IL-4 secretion; this was significantly higher than IL-4 detected in cultures containing either mismatched MSC at this ratio. With the exception of partially mismatched MSCs at a ratio of 1:10, all MSCs increased IL-10 production by stimulated T cells after 24 hours (Figure 2D).

The ratio of Th1:Th2 cytokine production was determined based on IFNγ and IL-4 levels. After 24 hours this Th1:Th2 ratio was reduced in cultures containing syngeneic and partially mismatched MSC demonstrating a shift towards Th2 production compared to controls; this was significant at the higher cell ratio of 1:1 (Figure 2E). There was no significant difference between Th1:Th2 ratio of T cells cultured with fully mismatched (BALB/c) MSCs compared to controls at either the 1:10 or 1:1 MSC:T cell ratio. However, there was a significant difference between the Th1:Th2 cytokine ratio of T cells co-cultured with BALB/c MSCs compared to that of either the syngeneic or partially mismatched cultures (Figure 2E).

In all groups, controls of un-stimulated CD4+ T cells with and without MSCs were included; no production of pro or anti-inflammatory cytokines was seen in these groups (results not shown).

### MHC mismatch influences the effect of murine MSCs on disease progression in CIA

**Figure 3:**
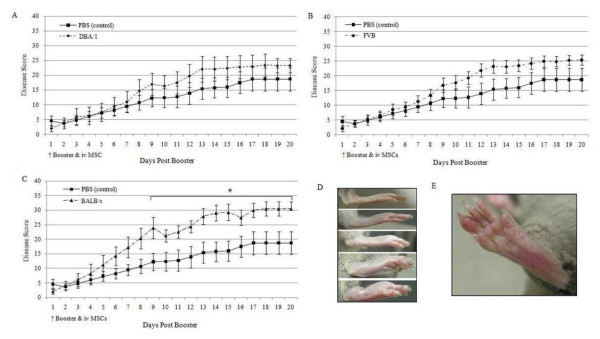
**The effect of systemically infused MSCs on the progression of CIA in DBA/1 mice**. **A**, Intravenous (iv) infusion of syngeneic DBA/1 MSCs at the time of booster immunization had no effect on disease progression. **B**, While there appeared to be some worsening of disease progression after iv delivery of partially mismatched FVB MSCs, this did not reach statistical significance. **C**, Systemic delivery of fully mismatched BALB/c MSCs resulted in a significant exacerbation of disease progression compared to control groups which received vehicle alone. **D**, Representative images of swollen hind paws ranging from normal (top image) to grossly swollen (bottom image). **E**, Representative image of dactylitis in a single digit. All experiments were performed with n = 12 and results are represented as mean ± standard deviation. *P <0.05, Shapiro Wilks testing for normal distribution followed by unpaired T and Mann Whitney U tests. CIA, collagen induced arthritis; MSC, mesenchymal stem cells; n, number.

**Figure 4:**
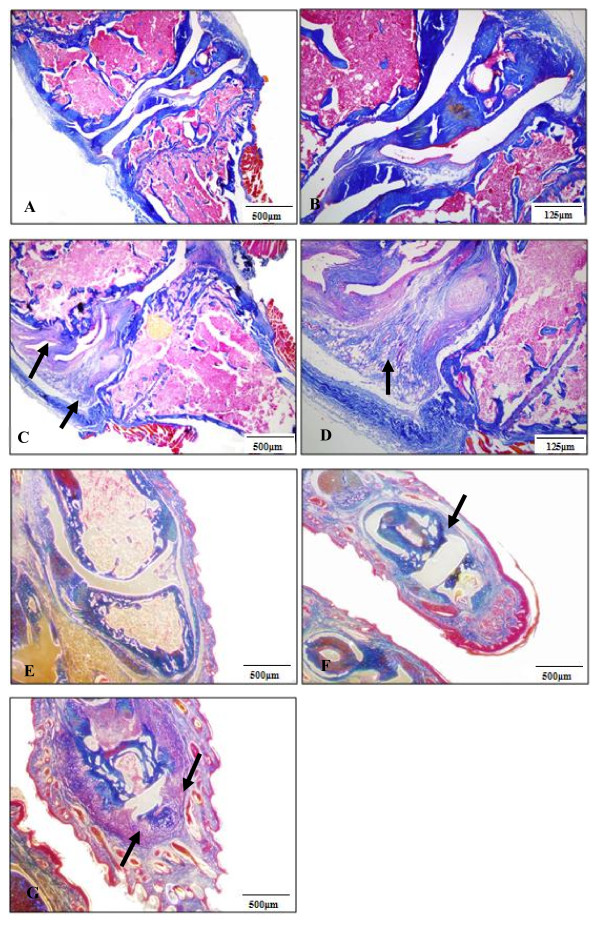
**Histological analyses of knee joints**. Fifty percent of the mice treated with vehicle control, syngeneic or partially mismatched MSCs had erosive disease compared to 70% of those treated with fully mismatched MSCs. **A, B**, A normal knee joint is illustrated (scale bar 500 μm and 125 μm, respectively). **C, D**, Synovial hypertrophy with erosive disease in the knee joint (black arrow) (scale bar 500 μm and 125 μm, respectively). **E**, A control interphalangeal joint (IPJ) of the paw (500 μm), **F**, IPJ with synovial hypertrophy and early erosive disease (black arrow) (500 μm) and **G**, a severely diseased IPJ with synovial hypertrophy and extensive erosive disease (black arrows); scale bars, 500 μm. MSC, mesenchymal stem cells.

CIA was induced in male DBA/1 mice by immunization with bovine IIC. Twenty-one days later a booster immunization was given along with an intravenous dose of MSCs, either syngeneic, partially matched or mismatched. Clinical scores were calculated based on the paw thickness across the metatarsophalangeal joints (Figure 3D) and dactylitis (Figure 3E); all mice developed obvious signs of arthritis. The systemic delivery of syngeneic MSCs had no effect on disease progression in CIA when compared to mice receiving vehicle control alone (Figure 3A). The delivery of H-2 matched allogeneic FVB MSCs appeared to worsen disease progression but this was not statistically significant (Figure 3B). However, infusion of allogeneic H-2 mismatched BALB/c MSCs resulted in significantly higher disease scores when compared to vehicle controls from day nine post-booster to the end of the experiment (Figure 3C). Histological evidence of erosive disease in the paw, ankle and knee joints of mice with CIA has been demonstrated previously [38,47]. In the present study knee joints were examined histologically to assess for inflammatory disease not evident on clinical examination and 95% of all knees analyzed had evidence of histological synovitis. Furthermore, erosive disease was demonstrated in 50% of mice receiving vehicle control, syngeneic or partially mismatched MSCs compared to 70% of mice receiving fully mismatched BALB/c MSCs (Figure 4B and 4D). This difference did not reach statistical significance and the mean clinical scores between erosive and non-erosive groups were similar (results not shown). Evidence of erosive disease was also found following histological examination of the interphalangeal joint of the paw (Figure 4F and 4G).

**Figure 5:**
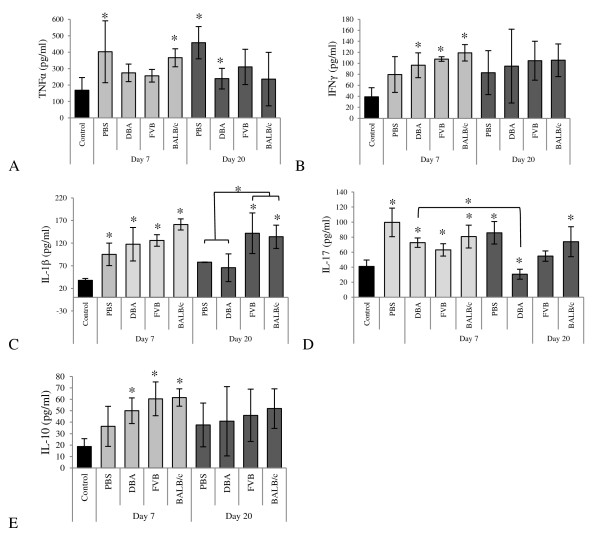
**Serum levels of pro-inflammatory cytokines**. **A**, At both 7 and 20 days after booster, and onset of CIA, TNFα levels were raised significantly in mice receiving vehicle alone (PBS) compared to pre-CIA control levels. TNFα levels were also elevated in mice treated with BALB/c MSCs at seven days. At day 20 TNFα levels were significantly suppressed only in DBA/1 treated mice compared to the PBS group. **B**, IFNγ levels were elevated in mice treated with any strain of MSC at seven days compared to pre-CIA controls. At 20 days there was a trend towards elevated IFNγ levels in all groups but this did not reach statistical significance. **C**, At seven days IL-1β was elevated in all groups, *P *<0.05 (2-way ANOVA). At 20 days elevated levels persisted in mice treated with allogeneic MSCs; this was significant compared to groups treated with either PBS vehicle or syngeneic MSCs, *P *<0.05 (2-way ANOVA). **D**, IL-17 levels were significantly elevated on day seven in all treatment groups. By day 20, IL-17 levels were significantly raised only in vehicle treated mice and those treated with fully MHC mismatched allogeneic MSCs (BALB/c). The level of IL-17 in mice treated with syngeneic MSCs was significantly lower at day 20 than all other treatment groups at this timepoint and also when compared to DBA/1 treated mice at day seven, *P *<0.05 (2-way ANOVA)**. E**, At day seven there was an increase in IL-10 levels in all groups treated with MSCs compared to untreated controls. This effect was not seen at day 20. There were 12 mice per treatment group and all measurements were assayed in triplicate, * *P *<0.05 (2-way ANOVA). Data represents mean ± standard deviation. ANOVA, analysis of variance; CIA, collagen induced arthritis; IFNγ, interferon gamma; IL-1β, interleukin 1 beta; IL-10, interleukin10; IL-17, interleukin 17; MHC, major histocompatibility complex; MSC, mesenchymal stem cells; PBS, phosphate-buffered saline; TNFα, tumor necrosis factor alpha.

Serum was collected 7 and 20 days after booster immunization and cytokine levels were analyzed. Serum TNFα levels were elevated in CIA mice treated with vehicle only compared to non-arthritis controls. There was no significant difference in TNFα levels between treatment groups or compared to vehicle controls (Figure 5A). At day seven levels of IFNγ were increased in mice receiving MSCs from all genetic backgrounds. This effect was transient and there was no significant difference between groups at 20 days (Figure 5B). At seven days IL-1β was elevated in all treatment groups compared to controls. This persisted in the serum of mice treated with either FVB or BALB/c MSCs. However, serum levels in mice treated with either PBS or syngeneic MSCs were lower than those treated with allogeneic MSCs (Figure 5C). IL-17 levels were elevated in all mice at seven days compared to non-arthritis controls and remained persistently elevated in mice treated with BALB/c MSCs. There was a highly significant suppression of IL-17 levels in the serum levels of mice treated with syngeneic MSCs over the course of the study (Figure 5D). Serum IL-10 levels were elevated only at the seven day time point in MSC treated mice compared to non-arthritic controls (Figure 5E). Serum IL-4 was not detectable at either 7 or 20 days (results not shown).

## Discussion

The ability of MSCs to suppress T cell proliferation and activation *in vitro *and their ability to promote repair suggest that they may be beneficial in treating inflammatory arthritis. However, studies using the CIA mouse model have been inconclusive with results varying from no benefit to exacerbation of disease [23,29-32]. Studies have used either MSC delivery at a single time point or multiple injections. We opted to use a single injection to directly compare the effect of single MSC strains and avoid confounding factors such as the development of an immune response to the MSC. Variations in the method of CIA induction, mouse strains used for MSC preparation, scoring systems, timing, cell number, and delivery makes it difficult to compare these published studies (Table 2) [48]. However, data suggest that any beneficial effect of MSCs may be mediated through the suppression of the Th1 and Th17 driven responses along with an increased antigen specific T regulatory cell response [40,48,49]. This study represents the first direct comparison of the effect of different murine MSC strains on the progression of CIA and does not demonstrate any protective effect of MSCs. Indeed, BALB/c MSCs clearly exacerbated disease both biochemically and clinically while FVB MSCs showed a trend towards increased disease severity with associated increases in host IL-1β production. Additionally, the disease scores seen in BALB/c treated mice were significantly higher than controls but did not reach statistical significance when compared to other MSC treated groups; this reinforces the lack of any amelioration of disease with MSCs and perhaps suggests a subtle subclinical negative impact on arthritis even in syngeneic MSC treated mice.

**Table 2:** Published studies of MSCs in CIA.

Author, Year	MSC	Route	Cell number	Day of delivery	Outcome
Djouad, 2005	C3H10T1/2	iv	10^6^	0	-
			10^6^	21	-
			4 × 10^6^	0	-
			4 × 10^6^	21	Exacerbation
Augello, 2007	BM C57/Bl/10 or GFP transgenic	ip	5 × 10^6^	21	Amelioration
Choi, 2008	BM DBA/1	iv	10^6^	21, 28, 35	Delayed onset
Gonzalez, 2009	hAdMSC Ad DBA/1 Ad C57/Bl6	ip	10^6^	5 days after disease onset	Amelioration
Schurgers, 2010	BM DBA/1 WT or IFNγ KO	iv or ip	10^6^	1, 16, 23	-

Ad, adipose derived; BM, bone marrow derived; h, human; ip, intraperitoneal; iv, intravenous; KO, knock out; WT, wild type.

Several studies have demonstrated the immune privileged status of MSCs [24,33]. However, others have suggested that allogeneic MSCs can elicit a host immune response [50,51] and genetic disparity reduced the therapeutic benefit of MSCs in rat models of ischemic heart disease [52]. In particular, recent data in rat heart allograft studies demonstrated the presence of activated T cells in secondary lymphatic organs following the administration of MHC mismatched MSCs suggesting that allogeneic MSCs may elicit an immune response [53]. The ability of MSCs to suppress T cell proliferation has previously been demonstrated with Carboxyfluorescein succinimidyl ester (CFSE) staining [54]. Here, we focused on the effect of MSCs on cytokine production and have demonstrated a superior ability of syngeneic MSCs to suppress T cell responses *in vitro *with a decrease in pro-inflammatory cytokine production and an alteration of the Th1:Th2 ratio. MHC mismatched allogeneic MSCs not only had less immunosuppressive effects *in vitro *compared to MHC matched cells but had a deleterious effect on disease progression *in vivo*. Fully mismatched BALB/c MSCs were the only cells which resulted in increased serum TNFα levels at day seven while all three stains of MSCs resulted in elevated serum IFNγ. By day 20 the effects on IFNγ production were lost while only syngeneic DBA/1 cells resulted in a significant suppression of TNFα production compared to mice treated with vehicle alone. It was also demonstrated that all three strains of MSCs augmented the production of both IL-1β and IL-17. This effect was sustained in mice treated with either allogeneic cell with respect to IL-1β while only fully mismatched BALB/c MSCs had the effect of persistently elevating IL-17 levels by day 20. Interestingly, this elevation in pro-inflammatory cytokines was significantly suppressed in mice treated with DBA/1 cells by day 20 after disease induction. Taken together these data demonstrate an early increase in systemic inflammation in mice with arthritis treated with MSCs which is mediated predominantly through IFNγ, IL-1β and IL-17. While treatment with syngeneic MSCs is insufficient to suppress pro-inflammatory cytokine production at seven days, with time they have the effect of suppressing both IL-17 and IL-1β secretion. This may relate to increased survival of the cells *in vivo *or, given the clinical worsening of disease, a true deleterious effect of allogeneic MSCs on arthritis progression in an inflammatory environment. It has been reported that pre-stimulation of MSCs with IFNγ may have the desirable effect of augmenting MSC immunosuppression *in vitro *[53]; however, it is clear that the inflammatory milieu of CIA does not facilitate this phenomenon. *In vivo *IL-4 levels were below detectable limits but a significant increase in IL-10 was demonstrated at day seven in all three treatment groups. While it has previously been shown that MSCs transduced to express IL-10 can ameliorate disease and the suppression of arthritis by adipose-derived MSCs was associated with increased synovial IL-10 levels [30,38], only a transient increase in this cytokine was demonstrated here. This was not sustained to 20 days and the impact of the expected MSC effect of increased IL-10 production was not sufficient to impact arthritis progression.

The conflicting results regarding the potential role of MSCs in arthritis have recently been highlighted [48]. By comparing MSCs from different murine strains in CIA under identical experimental conditions, these data go some way to addressing the conflicting results in previous studies. It has been demonstrated that, despite their low immunogenicity, the genetic mismatch between the MSC and host has a significant impact on the ability of MSCs to alter disease progression. Furthermore, the effect of MSCs on disease progression appears to be mediated predominantly by alterations in host IL-17 and IL-1β production.

## Conclusions

The potential of MSCs to treat conditions such as arthritis may help us to meet the challenge of inducing remission and joint regeneration in patients with disease refractory to conventional treatment options. However, before translation to the bedside it is of fundamental importance that we fully understand pre-clinical data. While CIA is a reliable model of RA which continues to be used successfully in the development of therapeutics, our data clearly demonstrate that a single injection of MSCs from different genetic backgrounds has disparate effects on disease progression as demonstrated in clinical scores and serum cytokine levels. This suggests that further studies looking at the effect of varying cell numbers and repeated delivery of MSCs are warranted in the future. These data need to be strongly considered when both analyzing and developing pre-clinical studies of inflammatory conditions if the transition of MSCs into clinical practice is to be successful.

## Abbreviations

ANOVA: analysis of variance; ASC: adipose derived stem cell; CD: cluster of differentiation; CEM: complete expansion medium; CIA: collagen induced arthritis; CII: type II collagen; (D)MEM: (Dulbecco's) modified Eagle's medium; ELISA: enyme-linked immunosorbent assay; FACS: fluorescence activated cell sorting; FBS: fetal bovine serum; GAG: glycosaminoglycan; IFNγ: interferon gamma; IL: interleukin; MHC: major histocompatibility complex; MSC: mesenchymal stem cell; PBS: phosphate-buffered saline; P/S: penicillin and streptomycin; RA: rheumatoid arthritis; Th: T helper cell; TNFα: tumor necrosis factor alpha.

## Competing interests

The authors declare that they have no competing interests.

## Authors' contributions

CS made significant contributions to study design, *in vitro *and *in vivo *experimental work, analysis of data, document preparation and submission. MM contributed to study design, analysis of data and document preparation. MG contributed to *in vitro *T cell stimulation studies. RP contributed to study design and *in vivo *data collection. CE contributed to study design, data analysis and document preparation. COF contributed to study design, histological analysis and document preparation. GS contributed to *in vitro *MSC work and flow cytometry. FB contributed to study design, data analysis and document preparation. All authors read and approved the final manuscript.
